# The thermal ecology of flowers

**DOI:** 10.1093/aob/mcz073

**Published:** 2019-06-17

**Authors:** Casper J van der Kooi, Peter G Kevan, Matthew H Koski

**Affiliations:** 1 Groningen Institute for Evolutionary Life Sciences, University of Groningen, Groningen, the Netherlands; 2 School of Environmental Sciences, University of Guelph, Guelph, Canada; 3 Department of Biology, University of Virginia, Charlottesville, VA, USA

**Keywords:** Abiotic effects, climate, evolution, fecundity, flower colour, heliotropism, pollination, morphology, reproduction, temperature

## Abstract

**Background:**

Obtaining an optimal flower temperature can be crucial for plant reproduction because temperature mediates flower growth and development, pollen and ovule viability, and influences pollinator visitation. The thermal ecology of flowers is an exciting, yet understudied field of plant biology.

**Scope:**

This review focuses on several attributes that modify exogenous heat absorption and retention in flowers. We discuss how flower shape, orientation, heliotropic movements, pubescence, coloration, opening–closing movements and endogenous heating contribute to the thermal balance of flowers. Whenever the data are available, we provide quantitative estimates of how these floral attributes contribute to heating of the flower, and ultimately plant fitness.

**Outlook:**

Future research should establish form–function relationships between floral phenotypes and temperature, determine the fitness effects of the floral microclimate, and identify broad ecological correlates with heat capture mechanisms.

## INTRODUCTION

Obtaining and maintaining an optimal flower temperature is often imperative for successful plant reproduction. Although some plants may actively dissipate excess heat from their reproductive organs (e.g. Patino and Grace, 2002), for many species the adaptations for absorbing exogenous heat are of vital ecological importance (Corbet, 1990). For example, for many species – particularly those in arctic or alpine regions and early spring ephemerals – small increases in flower temperature enhance reproductive success (Stanton and Galen, 1989; Corbet, 1990; Ida and Totland, 2014; Distefano *et al.*, 2018). The temperature at which pollen germinates and pollen tube growth is optimized has also been proposed to be a predictor of plant species distributions (Rosbakh and Poschlod, 2016).

Both the plant and anthophilous insects can benefit from a flower temperature that differs from ambient conditions. Many metabolic processes related to plant reproduction are temperature-dependent; for example, low temperatures impede plant cell division and expansion (e.g. Körner, 1998). A suite of pre- and post-fertilization processes also require an optimal temperature (reviewed by Rosbakh *et al.*, 2018). As with most relationships between temperature and developmental rates, pollen germination and tube growth generally display a unimodal response to temperature, declining in extreme thermal environments (Steinacher and Wagner, 2012; Peng *et al.*, 2015; Distefano *et al.*, 2018). For example, experimental heating of early spring-flowering *Helleborus foetidus* flowers led to an increase in pollen tube number (Herrera and Medrano, 2016). Obtaining and maintaining the optimal flower temperature is also important for embryo formation and abortion (Stephenson, 1981). Finally, temperature mediates the synthesis of components of floral scent and its vaporization (Sagae *et al.*, 2008). Thus, temperature modulation at the level of the flower is crucial for maintaining pollen, ovule and seed viability, as well as emission of pollinator-attracting volatiles.

Exogenous heat absorption may, in addition to providing direct benefit to the flower’s reproductive organs, also benefit pollinators and thus indirectly impact the plant’s reproductive success. We identify at least two ways by which warm flowers may promote visitation by pollinators. First, insects can elevate their body temperature by basking in flowers (e.g. Hocking and Sharplin, 1965; Kevan, 1975; Heinrich, 1979; Herrera, 1995), and when flowers offer greater heat rewards this promotes their reproductive success. For example, the arctic *Dryas integrifolia* flowers are on average more than 7 °C warmer than the ambient air, and the insects that visit them can be up to 15 °C warmer (Kevan, 1975). Bees are able to detect differences in flower temperatures, which has important consequences for their floral preferences and pollination behaviour. Bees were shown to prefer flowers with experimentally warmed nectar over those with unheated nectar (Dyer *et al.*, 2006), and this preference is stronger with decreasing ambient temperatures (Norgate *et al.*, 2010). Recently, using thermography Harrap *et al.* (2017) showed that 55 % of plant species displayed within-flower temperature differences of at least 2 °C that can be detected by bees, highlighting that the significance of flower temperature for visitation by pollinators may be widespread. Second, warm flowers may be preferred by pollinators, because they offer more and/or higher quality rewards compared with low-temperature flowers. For example, nectar volume and sugar production have been shown to increase up to a temperature of 38 °C (Petanidou and Smets, 1996). By the same token, we expect that – as with many biochemical reactions – elevated temperatures could increase protein and fat synthesis in the theca, though the effect of temperature on pollen nutrition has not yet been investigated experimentally. Given that bees select the highest nutritional value food when provided the opportunity (Ruedenauer *et al.*, 2015), they may select for warm flowers when these flowers offer more nutritional reward. In summary, plants with slightly elevated flower temperatures may experience a fitness advantage due to both elevated (chemical and/or physiological) processes inside the flower as well as increased visitation by pollinators.

**Fig. 1.**
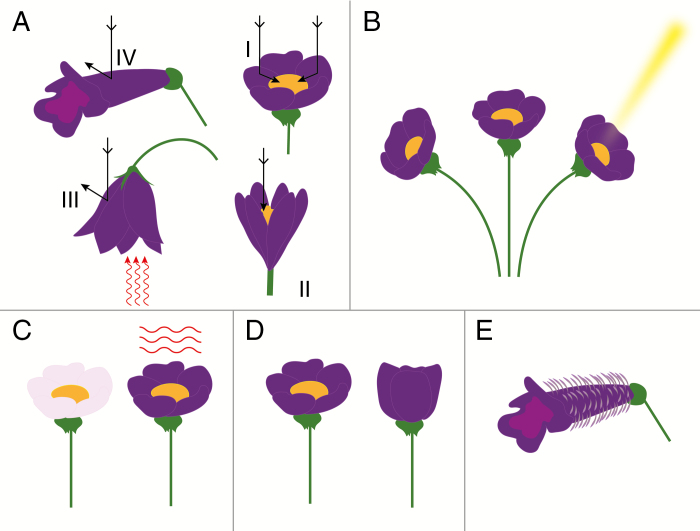
Floral attributes that increase exogenous heat capture. (A) The flower’s shape determines the amount of heat captured and retained. In upward-facing disc-, bowl- or bell-shaped flowers (I, II), the reproductive organs can heat under direct sunlight and through additional reflection of light by the petals. For pendant, hanging flowers (III), the reproductive organs capture little direct sunlight, but the flower may entrap heat radiated from below, and reproductive organs are less exposed to wind and rain. For tubular flowers (IV), relatively little direct sunlight reaches the reproductive organs, but the (partially) enclosed inner chamber may have an increased temperature due to microgreenhouse-like effects. (B) The orientation of flowers determines the immediate capture of sunlight. Via changes in the flower’s orientation (e.g. heliotropism), the amount of heat captured can be maximized over the course of day. (C) Darkly coloured flowers may absorb more light that can be re-emitted as heat, although the role of colour in modification of the floral thermal environment seems to be highly system-specific. (D) Flower opening–closure behaviour can protect the reproductive organs from exposure to extreme temperatures, wind or rain. (E) Pubescence increases the boundary layer of the flower, working as an insulation layer and increasing heat retention.

A suite of factors enables flowers to obtain and maintain the optimal thermal balance (Figure 1). (1) A flower’s shape and size largely predict the amount of light captured by the perianth and reproductive organs. For example, a sun-facing daisy maximizes heat capture, but for flowers with a relatively narrow opening such as tubular flowers the amount of radiant heat that enters the flower is limited. (2) The orientation of a flower determines the size of the sun-facing floral area and is thus correlated with the amount of heat energy absorbed. Flower movements, especially heliotropism (‘solar tracking’), importantly modify the flower’s orientation and affect heat capture. (3) The flower’s coloration may modify the absorption of heat. Very dark flowers may have higher intra-floral temperatures because they absorb more energy than very light and reflective flowers (e.g. Jewell *et al.*, 1994; McKee and Richards, 1998). (4) A flower’s opening and closure behaviour (nyctinastic movements) can shield the reproductive organs from exposure to excessive heat or cold (van Doorn and van Meeteren, 2003). Flower closure may also protect the reproductive organs from moisture (Von Hase *et al.*, 2006), which increases heat loss. (5) Flower pubescence may increase heat retention. (6) Endogenous heat production (thermogenesis) is a relatively rare feature of plants generating their own heat. The different aspects of flower temperature modulation have been studied for over a century (e.g. Kerner von Marilaum and Oliver, 1895; Büdel, 1956; Corbet, 1990), but there is no up-to-date overview of studies on this topic.

In this review, we synthesize current knowledge on the various mechanisms by which plants modify flower temperature. Specifically, we discuss six key aspects of flowers that help to increase their temperature: flower shape, orientation (including heliotropism), colour, opening and closure movements, pubescence and thermogenesis. We exclude the exceptional cases of heat production by other organisms such as nectar yeasts (Herrera and Pozo, 2010). Where possible, we provide quantitative estimates of how different floral attributes contribute to flower temperature. Throughout the text we highlight open research questions and suggest approaches that will push the bounds of our knowledge of the thermal ecology of flowers.

## FLOWER MORPHOLOGY

Floral shape and size influence heat accumulation and retention within flowers. First, floral shape can determine the direction from which radiant and convective heat enter the corolla, and size determines the surface area that can absorb radiation. Second, the degree to which the corolla encloses the reproductive structures determines the degree to which the organs are subject to ambient thermal conditions. Based on how floral shape influences thermal dynamics, Kevan (1989) categorized flowers into six primary groups. Here, we distil those groups down to four based on shared morphology or function: (1) disc- and bowl-shaped flowers, (2) inverted bells, (3) hanging bells and (4) ‘microgreenhouses’ (Figure 1A). Whereas microgreenhouses appear to be restricted to arctic or alpine regions, the remaining categories can be found in all bioclimatic regions. In this section, we summarize the features of these groups with respect to modification of the floral thermal environment and discuss what is known about the effect of shape on flower temperature. We then propose how quantitative (in addition to categorical) and multivariate metrics of floral morphology will provide a deeper understanding of how floral shape impacts floral temperature.

Bowl- or disc-shaped flowers (see case I in Fig. 1A) are generally actinomorphic, with reproductive structures that are largely exposed to ambient conditions. These flowers often resemble the shape of a paraboloid dish, with incident light reflected towards the centre of the flower where the reproductive organs are located (Kevan, 1975; van der Kooi *et al.*, 2017). Common disc-shaped inflorescences occur in families such as Asteraceae, Rosaceae and Hypericaceae, and more bowl-shaped flowers in the Ranunculaceae or Papaveraceae. Indeed, experimental removal of petals of *Dryas integrifolia* and *Saxifraga oppositifolia* reduced the flower’s temperature excess by about 70 % (from 6.5 to 2 °C and from 2.5 to 0.5 °C, respectively; Kevan, 1970), highlighting the petal’s importance for flower temperature.

Exogenous heat capture in bowl-shaped flowers depends on the angle of solar illumination in a cosine fashion (Kevan, 1970; Stanton and Galen, 1989; Totland, 1996), with flower aperture and tubularity being important predictors. In fairly flat flowers such as those of *Dryas integrifolia* (Rosaceae), temperature excesses due to exogenous heat absorption are rather constant for a wide window of illumination angles, i.e. within 60° of directly facing the sun (Kevan, 1970). For the more tubular flowers of *Saxifraga oppositifolia* (Saxifragaceae), increases in temperature occur under a smaller window of illumination angle, i.e. within <30° of directly facing the sun (Kevan, 1970). However, for other species, such as *Ranunculus acris* (Ranunculaceae), the correlation between orientation angle and heat capture is more variable and whether the relationship is linear or cosine is unknown due to large variation (Totland, 1996). How changes in tubularity and aperture of the flower correlate with heat capture requires more systematic investigation.

In disc- and bowl-shaped flowers, anthers and ovules are subject to exposure by direct sunlight, wind and rain, and therefore these species are more likely to feature attributes such as flower movement (e.g. heliotropism), petal opening and closure, and modification of pigmentation to regulate floral temperatures (see sections on orientation, colour and opening/closing of flowers). Indeed, most species with documented heliotropism have disc- or bowl-shaped flowers (see ‘flower Orientation and Heliotropism’). In *Argentina anserina* (Rosaceae), ultraviolet-absorbing pigmentation (the ‘UV bulls-eye’) of the bowl-shaped flower may modify internal reflection of harmful UV light onto the anthers (Koski and Ashman, 2015). Although work in *A. anserina* deals with the effects of UV light on pollen viability rather than temperature, it indicates that flower shape has the potential to modify the abiotic environment of the flower.

Flowers shaped that resemble ‘inverted bells’ (Kevan, 1989) have the corolla opening facing upward similar to disc and bowl flowers, but are more tubular in form (see case II in Fig. 1A). Common species in this category are in the families Saxifragaceae, Gesneriaceae, Brassicaceae and Caryophyllaceae. In inverted bell flowers, the angle of insolation is expected to have a stronger effect on floral temperature than in disc-shaped flowers (Kevan, 1989) and these taxa are more likely to trap heat energy (McKee & Richards, 1998). We propose that two additional traits may affect floral temperature in inverted bells and warrant investigation. First, the degree of corolla flare in inverted bells is variable among species, and could determine the area of the flower that is capable of absorbing heat. Second, the degree of anther and/or stigma exertion can vary, from those housed deep in the corolla tube to those exerted well beyond the corolla. The degree of anther and stigma exertion may influence the temperature experienced by pollen and stigma, and thus the magnitude of selection to modify floral temperature.

‘Hanging bell’ flowers are those in which the corolla opening faces the ground (case III in Fig. 1A) and occur in species of *Galanthus* (Amaryllidaceae), *Campanula* (Campanulaceae) and *Erica* or *Vaccinium* (Ericaceae) (Büdel, 1956; Kevan, 1989). Hanging bells may acquire heat from ground radiation, and thus the angle of corolla opening in relation to the ground could have an important influence on floral temperature. Some arctic hanging bell species can be 3–11 °C above ambient temperature (Kevan, 1989), but few studies have evaluated floral thermal environments in these types of flowers. Hence, the relative contributions of overall shape, physiology and colour remain unknown. The ratio of heat captured from the ground versus that acquired from direct capture of sunlight for hanging bells also remains unknown.

Some flowers function as ‘microgreenhouses’ based on their morphology that traps heat and re-radiates it within in a (partially) enclosed chamber (case IV in Fig. 1A) (Kevan, 1989; Corbet, 1990). The Himalayan herbs *Rheum alexandrae* and *R. nobile* (Polygonaceae) are arguably the best-studied cases of microgreenhouses. These species produce large many-flowered racemes that are covered by large translucent bracts (Omori *et al.*, 2000). The bracts effectively filter light in the UV range, but transmit longer wavelengths, resulting in up to a 10 °C increase of the plant’s interior on sunny days (Song *et al.*, 2013). Elevated temperatures enhance pollen germination and pollinator visitation, and the microgreenhouse anatomy further provides a stable interior humidity (Song *et al.*, 2015).

Other examples of microgreenhouse effects have been identified in *Saussurea velutina* (Asteraceae) and *Pedicularis arctica* (Orobanchaceae). Removal of bracts that cover the inflorescences led to an average reduction of 2.5 °C, with outliers to 16 °C in *S. velutina* (Yang and Sun, 2009). In *P. arctica*, flowers in full sun have temperature excesses of over 6 °C (Kevan, 1970), although the relative contributions of orientation to the sun and shielding of reproductive organs by the perianth have not been disentangled. Comparative anatomy on *Rheum* and *S. velutina* leaves and translucent bracts shows that bracts are thinner, have fewer cells per area than leaves and have less chlorophyll and carotenoid pigment (Omori *et al.*, 2000; Tsukaya, 2002; Yang and Sun, 2009). Reflection of light occurs at boundaries of media with different refractive indices, such as water and cell walls (van der Kooi *et al.*, 2016; van der Kooi and Stavenga, 2019), so a reduction in the amount of cell structures decreases reflection and thereby increases light transmission.

Microgreenhouse-like effects may occur also in other structures, outside the centre of the flower. *Silene* spp. (Caryopyllaceae) have a microgreenhouse-like morphology with inflated chamber-like calyces, and some of these types of flowers can also be associated with pubescence that provides insulation (see ‘Flower Pubescence’). Similar to flowers, hollow stems may also feature a microgreenhouse-like effect, where accumulation of heat trapped within the stem leads to an increase in interior temperature (Kevan *et al*. 2018, 2019), which may enhance development of the flower bud immediately above. In addition to the transparency of floral parts, one-way mirroring effects have been proposed to further increase translucency and capture incident solar radiation (Kevan, 1989), although physiological observations of such structures supporting this claim have, to our knowledge, not been put forward.

Although flower morphology has been noted to influence flower temperature (Kevan, 1989), to our knowledge only one study has conducted a formal statistical test of the impact of flower shape on temperature for many species at once. Shrestha *et al.* (2018) measured ambient and floral temperature for 30 species of the Australian flora categorized by shape. Floral shape did not significantly impact the deviation of floral temperature from ambient conditions (Shrestha *et al.*, 2018), although tests taking into account phylogenetic relatedness were not performed. It should be noted that Shreshtha *et al*.’s (2018) study was conducted in temperate Australia, and the relationships between floral temperature modulation of floral morphology have largely been studied in arctic or alpine regions. Treating floral shape as a discrete character, however, may not adequately capture the relationships between morphology and floral temperature, given the many ways by which flower shapes vary.

We propose that future studies consider quantitative measurements that incorporate various aspects of floral morphology, and that the selection of traits should be guided by specific predictions about how shape parameters should impact thermal dynamics. (1) The direction of the corolla opening should be measured because it impacts the direction and type of radiation received by the flower. (2) The size of the corolla opening should influence the rate of heat loss. (3) Overall metrics of floral size should be taken because size should correlate with the area of thermal absorption and heat loss. (4) The degree of anther and stigma exertion may determine the selection to maintain an optimal floral temperature. (5) Multivariate approaches incorporating multiple floral traits would help determine which aspects of floral morphology are most influential on floral temperature. Although this list of characters is not exhaustive, we hope that it provides examples of how form and function relationships should be considered when choosing shape parameters to be measured.

The most amenable approach to understanding the impact of flower shape and size on floral temperature in natural flowers is an interspecific comparative one, because large-scale variation in shape within a species is less common. Modelling as well as experimental approaches with three-dimensional printed flowers, where different aspects such as flower aperture, curvature and size can be modified individually or together (e.g. Peng *et al.*, 2019), could help to relate floral morphology to the flower’s thermal balance. Finally, detailed anatomical and physiological studies on real flowers could help to improve our understanding of light transmission and (convective) heat transfer.

## FLOWER ORIENTATION AND HELIOTROPISM

The orientation of flowers is known to be important for their visibility to and visitation by pollinators (Fenster *et al.*, 2009; Yon *et al.*, 2017), but the orientation relative to the sun (Fig. 1B) is also important for the flower’s thermal balance. In open habitats, the reproductive organs of upward-facing flowers capture the most sunlight. Pendant, downward-facing flowers are less likely to capture direct sunlight, although they may capture convection heat from the ground (see ‘Flower Morphology’).

Solar tracking, or (dia-)heliotropism of flowers as defined by Darwin, is a phenomenon that has intrigued scientists for decades and is considered to contribute to the warming of flowers, especially in the Arctic (Kevan, 1975; Stanton and Galen, 1989; Luzar and Gottsberger, 2001). As opposed to phototropism where the plant organ gradually grows towards the light, in the case of heliotropism, the organ’s orientation is adjusted continuously over the course of a day. Phototropism is categorized as heliotropism when it occurs quickly. Flower heliotropism occurs in at least 17 genera from seven plant families (Supporting Data Table S1); this widespread occurrence illustrates that it evolved multiple times independently and probably has functional significance.

Flower heliotropism increases the temperature of the reproductive organs and/or that of basking insects (e.g. Kevan 1972, 1975; Galen, 2006; Zhang *et al.*, 2010; summarized in Table S1), although the impact of heliotropism on floral temperature may vary with ambient temperature or population (Krannitz, 1996; Totland, 1996). In nine out of ten species for which the effects of flower heliotropism on temperature were experimentally investigated, it was found that heliotropism provides benefits for the flower’s thermal balance and increases reproductive output (Table S1). Benefits of heliotropism include, but are not limited to, increased gynoecial temperatures, heavier seeds and more visits by insects. It is important to bear in mind, however, that these results may be subject to publication bias, i.e. positive results – papers reporting an effect of heliotropism are more likely to be published than negative results. Nevertheless, for at least one Ranunculaceae species (*Ranunculus acris*), it was shown that flower heliotropism does not provide clear benefits for the plant’s reproduction (Totland, 1996).

For many heliotropic flowers in which temperature excesses were reported, heliotropism was assumed to be the primary reason, but this conclusion was not completely justified (Table S1). Indeed, in full sun, non-heliotropic flowers can be warmer than neighbouring heliotropic flowers (e.g. Rejšková *et al.*, 2010), because the inflorescence architecture and colour differ and not because of the differences in heliotropism per se. Thus, detailed experiments, such as tethering flowers (Stanton and Galen, 1989; Galen and Stanton, 2003) are required to understand the importance of the flower’s orientation. It is currently impossible to calculate an average effect of the contribution of heliotropism on the flower’s overall temperature (Serrano *et al.*, 2017), because heliotropic species differ greatly in flower size, form and colour, as well as in phylogenetic ancestry and geographical distribution. In other words, the biological significance of heliotropism is condition- and species-specific.

Understanding the mechanisms behind heliotropism may help to elucidate how and under what circumstances it evolved, and ultimately contribute to understanding its biological function. The mechanistic underpinnings of flower heliotropism have been studied in a few species, revealing both similarities and differences (Stanton and Galen, 1993; Sherry and Galen, 1998; Zhang *et al.*, 2010; Atamian *et al.*, 2016). Sunflowers (*Helianthus* spp., Asteraceae) are arguably the best-known examples of heliotropism, although – contrary to popular belief – heliotropism in sunflowers ceases at the onset of anthesis, leaving the flower heads in a fixed eastward orientation. Atamian *et al.* (2016) found that sunflower heliotropic movements result from differential stem elongation induced by a moving gradient of auxin. During the day, the auxin gradient shifts from west to east, causing increased growth on the shaded side, causing the stem to bend towards the illuminated side. Stem elongation on the shaded side also underlies heliotropic movements in flowers of *Ranunculus adoneus* and *Anemone rivularis* (Ranunculaceae) (Stanton and Galen, 1993; Sherry and Galen, 1998; Zhang *et al.*, 2010), but the flower organ responsible for photoreception is different. In *R. adoneus* heliotropic movements are mediated by blue light received by the apical region of the peduncle (Stanton and Galen, 1993; Sherry and Galen, 1998), but in the related *A. rivularis* and in *Dryas octopetala* (Rosaceae), the tepals or petals mediate heliotropism (Corbett *et al.*, 1992; Zhang *et al.*, 2010). Thus, even in related species, heliotropism appears to have evolved in different ways.

Flower heliotropism appears to be particularly prevalent in arctic and alpine regions (Table S1), but whether it actually occurs more frequently there or whether this observation is due to ascertainment bias remains unknown (see also van der Kooi, 2016). Although floral heliotropism appears to be widespread throughout the Angiosperms, its presence can vary within a genus: *Viola pedunculata* is heliotropic (Kutschera and Briggs, 2015), whereas other *Viola* species are not (Bernhardt *et al.*, 2016). Comparative analyses or reciprocal transplantation studies that associate the presence of heliotropism with geographical and bioclimatic parameters will be useful for understanding the ecological factors and floral characteristics that shape variation in heliotropism.

We identified at least four potential costs for flower heliotropism, but only one has been tested so far. (1) Increased water loss via transpiration may impose a cost given the increase in the flower’s temperature that is associated with heliotropism. Galen (2006) examined water loss due to heliotropism in *R. adoneus* and found that sun-tracking flowers required 29 % more water than flowers that were not tracking the sun – this increase is probably due to transpirational cooling of the hotter flowers. (2) Flowers that are aligned with the sun constantly may suffer from increased UV light and high UV exposure may be particularly detrimental for pollen performance (e.g. Demchik and Day, 1996; Wang *et al.*, 2010; Koski and Galloway, 2018). (3) Heliotropic growth probably requires extra energy and nutrients compared to non-heliotropic stem elongation, alhough the energy requirement for heliotropism has never been quantified. (4) The mechanistic underpinnings of heliotropism (differential cell growth, expansion and/or turgor) may undermine the mechanical strength of the peduncle. The latter two costs are, at this point, speculative, and require experimental validation. Regardless, all potential costs could act as constraints to the evolution of heliotropism.

The importance of floral orientation with respect to precipitation and accessibility to pollinators has received considerable attention in the last few decades (Aizen, 2003; Fenster *et al.*, 2009; Lin and Forrest, 2019), whereas few studies have focused on temperature effects related to orientation. For anthesed sunflower heads and dark-flowered *Oncocyclus* flowers, eastward orientation is thought to increase morning flower temperature, which may enhance pollinator attraction (Sapir *et al.*, 2006; Atamian *et al.*, 2016), although there may be other non-mutually exclusive explanations. For sunflowers for example, eastward-facing flower heads could benefit from increased evaporation of morning dew, reduced fungal growth, decreased (excessive) radiant heat capture during midday and/or enhanced maturation of fresh, new florets in the morning sun (Lang and Begg, 1979; van der Kooi, 2016). The flowers of *Nicotiana attenuata* (Solanaceae) show a rhythmic vertical change in orientation throughout their 2- to 3-d lifespan: at dusk, flowers open and move upwards, and at dawn, flowers close and move downwards (Yon *et al.*, 2017). The upward-facing orientation helps probing by hawkmoths and increases pollination success (Yon *et al.*, 2017), but a recent study by Haverkamp *et al.* (2019) showed that the downward orientation during daytime also shields the pollen from excessive heat. Experimental simulation of daytime temperatures showed that the downwards orientation yields a 7 °C reduction in within-flower temperature compared to tethered upward-facing flowers, which is beneficial for pollen germination (Haverkamp *et al.*, 2019).

Finally, there are reports of species that feature a non-random flower orientation, such as facing the sun at noon, but are not heliotropic. Studies on flowers of *Ipomoea pes-caprae* and *Merremia borneensis* (both Convolvulaceae) that exhibit an approximately sun-facing orientation during midday showed that this probably contributes to the gynoecial temperature and increases visitation by pollinators (Patiño *et al.*, 2002). This phenomenon, for which the term ‘seasonal heliotropism’ has been coined (Patiño *et al.*, 2002), is likely to be more widespread than generally appreciated. As an example, in temperate climates, we noted that inflorescences of a variety of species (e.g. *Bellis perennis* and other Asteraceae) seem to bend to the sun during midday, especially when ambient temperatures are low (our personal observation). Although this sun-facing orientation may not be striking as for heliotropic species and could simply be due to phototropism, such non-random distributions of inflorescence orientation hints at an adaptive function and deserves further study.

## FLOWER COLORATION

The colours of flowers are considered to have evolved primarily with respect to the visual capabilities of their pollinators (e.g. Chittka and Menzel, 1992), but colour may also play a role in temperature modification. An intuitive role of colour with respect to flower temperature is that darker colours absorb more radiant energy that can be converted to heat, thereby increasing the flower’s temperature (Fig. 1C). Apart from the direct physiological effects of colour on the flower’s temperature, bees can learn to associate a specific colour with temperature (Dyer *et al.*, 2006), meaning that colour may indirectly increase pollination success via the learning behaviour of pollinators.

Plasticity in colour as a response to temperature can modify floral temperature and provide substantial reproductive benefits. A series of experiments on *Plantago* (Plantaginaceae) revealed an intriguing relationship between the colour of the flower-bearing spike and its temperature. Plants that develop at low temperatures produce darker spikes that are 0.2–2.6 °C warmer in full sun. Floral colour plasticity (Lacey and Herr, 2005; Anderson *et al.*, 2013) is due to regulation of anthocyanin pigments (Stiles *et al.*, 2007). Plasticity in spike colour in response to temperature of development exists in the majority of *Plantago* species (Anderson *et al.*, 2013) and it has evolved in response to relative exposure to low temperatures (Lacey *et al.*, 2010). Similar anthocyanin plasticity has been shown as a response to light levels, with elevated anthocyanin production in full light conditions (Del Valle *et al*., 2018). Some of this response could have been due to elevated temperatures associated with increased solar radiation.

A few studies that have examined floral temperature in species with colour polymorphisms found contrasting results, suggesting no clear relationship between colour and temperature. For the arctic poppy, *Papaver radicatum* (Papaveraceae), yellow flowers are ~1.5 °C warmer than white flowers, and yellow-flowered plants are more frequent in colder regions (Mølgaard, 1989). In the flowers of *Lotus corniculatus* (Fabaceae), the keel encloses the reproductive organs and can be yellow- or brown-coloured. On cool sunny days, dark-keeled flowers can be almost 6 °C warmer than light-keeled flowers, but whether there is an association between colour morph frequency and climate is unknown (Jewell *et al.*, 1994). Purple-coloured flowers of *Ranunculus glacialis* (Ranunculaceae) are warmer and produce more seeds than white-coloured flowers (Ida and Totland, 2014). By contrast, in several colour polymorphic species there are no temperature differences between colour morphs (McKee and Richards, 1998; Sapir *et al.*, 2006; Mu *et al.*, 2010; Kellenberger *et al.*, 2019). Indeed, a recent study by Shrestha *et al.* (2018) on 30 Australian plant species suggested that there is no association between colour and temperature. A missing piece in studies on the relationship of colour with regard to the flower’s thermal regime is detailed knowledge on the flower’s anatomical and optical properties. Hence, it remains unknown whether the different colour morphs differed solely in pigmentation, or also in anatomy (e.g. van der Kooi *et al.* 2016, 2019) and how light rays and convective heat propagate inside the flower.

Although the relationships between petal coloration and floral temperature have been investigated, we are much further behind in our knowledge on the impact of pollen coloration on heat absorption and tolerance of thermal extremes by the male gametophyte. The colour of pollen can vary within and among species (Lunau, 2000; Koski and Galloway, 2018) and, like petal colour, darker pollen may have the capacity for elevated thermal absorption. Darker anthocyanin-based pigmentation may also be associated with elevated production of flavonol compounds that are crucial for pollen germination and pollen tube elongation (Mo *et al.*, 1992), suggesting that pollen pigmentation may influence the viability of pollen under extreme abiotic conditions. To our knowledge only one study has exposed different pollen colour morphs to extreme temperatures and evaluated pollen germination success. In American bellflower, *Campanula americana*, exposure to high temperatures reduced pollen viability of lighter coloured (white to light purple) pollen but not of dark purple pollen (Koski and Galloway, 2018). Thus, pollen pigmentation impacts thermal tolerance, but the mechanism(s) of protection from thermal stress have not yet been elucidated.

In addition to pigmentation, the structure of the petal surface and interior influences floral coloration and can play a role in absorption of heat. Flat and smooth surfaces may enhance within-flower reflection. *Ranunculus* and *Ficaria* flowers (both Ranunculaceae) feature a special anatomy that includes a flat and smooth surface, which renders them exceptionally glossy (van der Kooi *et al.*, 2017). Bearing in mind that several species of *Ranunculus* and *Ficaria* feature bowl-shaped flowers that are heliotropic – especially at lower temperatures (Stanton and Galen, 1989; Totland, 1996; Luzar and Gottsberger, 2001; Galen and Stanton, 2003) – the glossy surface enhances light reflection to the reproductive organs (van der Kooi *et al.*, 2017). For the Arctic poppy, *Papaver radicatum*, calculations by Kevan (1975) suggested that combined heliotropism and internal reflections can contribute as much as a 25 % to the flower’s heat budget, although experimental tests are lacking.

More studies that directly measure the relationships between flower colour, temperature and reproductive fitness are needed. Integrative approaches that determine the biochemical and structural aspects of coloration, and how these interact with shape and orientation parameters to modulate temperature will help to determine the relative effect of colour on the thermal dynamics of flowers. Additionally, trait manipulation studies would be useful to isolate the effects of colour relative to other traits on floral temperature. As floral pigmentation can be plastic in response to the thermal environment, quantification of temperature-induced plasticity in flower colour may help to disentangle how ambient temperature drives pigmentation changes and how this phenotypic change can in turn impact floral microclimates. Quantifying temperature-mediated selection on flower colour using selection analyses in natural populations will be crucial for determining the strength of selection by the thermal environment relative to other factors such as pollinators. Finally, comparative approaches that evaluate broad biogeographical patterns of flower colour distributions among species and plant communities (e.g. Koski and Ashman, 2016) will help to generate hypotheses regarding the function of flower colour with respect to floral microclimate.

## FLOWER OPENING AND CLOSURE

Floral opening and closing through petal movement (nyctinasty; Fig. 1D) is common across the plant kingdom, particularly in bowl- or disc-shaped species, and is often a physiological response to abiotic signals (e.g. light levels, humidity and temperature; reviewed by van Doorn and van Meeteren, 2003). Opening and closure of flowers occurs in families such as the Asteraceae, Gentianaceae, Geraniaceae, Iridaceae, Malvaceae, Ranunculaceae and Rosaceae (Andrews, 1929), suggesting it is a taxonomically widespread phenomenon. In an overview covering more than 100 species, Stirton (1983) found that night-time closure of inflorescences is widespread in the Asteraceae. Opening and closure can take several minutes or hours depending on the species. The majority of species (58 out of 106, i.e. 54 %) feature night-time closure (Stirton, 1983). Counterintuitively, some species fold their ligules downwards, so that they surround the peduncle and the carpel is left exposed, but whether this has an adaptive function (e.g. increasing heat retention in the capitulum) remains to be studied.

The proximate causes and mechanisms of nastic petal movements are relatively well studied (reviewed by van Doorn and van Meeteren, 2003; van Doorn and Kamdee, 2014), but almost nothing is known about the factors other than pollination that exert selective pressure on the timing of opening and closure, or the consequences of nastic petal movements for plant reproductive fitness. Differential turgor pressure or cell growth, for example due to an increase in growth rate at one side of the petal, can cause the flower to open or close. Flower closure is hypothesized to play a protective role against pollen damage from precipitation or desiccation that can render the pollen inviable (Von Hase *et al.*, 2006; van Doorn and Kamdee, 2014). Closure during rain also reduces pollen wash (Bynum and Smith, 2001; Abdusalam and Tan, 2014).

Conceivably, petal movement has the potential to modify floral temperature through both the retention of heat when petals close, and the release of heat when petals open. In the early spring-flowering *Crocus discolor* (Iridaceae), experimental inhibition of nocturnal flower closure resulted in reduced pollen viability, but the relative importance of humidity, nocturnal pathogens and flower temperatures cannot be disentangled (Prokop *et al.*, 2019). Zhang *et al* (2010) showed that temperatures of *Anemone rivularis* (Ranunculaceae) flowers with tepals removed vary slightly more in temperature, especially at night, suggesting that folding tepals around the reproductive organs have a shielding function. Similarly, tepals of *Tulipa iliensis* (Liliaceae) close during periods of low temperature and this maintains a more constant thermal environment within the flower (Abdusalam and Tan, 2014). In addition, the angle of petals relative to the sun modifies the amount of light reflected by the petals onto the anthers and ovules, including long wavelengths carrying heat energy, meaning that partial closure – even during the day – can have significant consequences for the flower’s thermal budget (Kevan, 1975; van der Kooi *et al.*, 2017). Temporal flower closure as a response to low temperatures and/or rain may be relatively common, especially in temperate areas and in early flowering species (our personal observation). If diurnal opening–closure does contribute to a more consistent thermal environment, we predict that nyctinastic petal movements should be more common in species that inhabit highly variable thermal environments relative to those that experience more constant temperatures.

To empirically test the effect of petal opening and closing on floral temperatures, future studies should use manipulative techniques that inhibit petal movements (e.g. Abdusalam and Tan, 2014) and compare internal floral temperatures between treatment and control groups. Moreover, to evaluate the fitness consequences of temperature differences, pollen and ovule viability should be assessed. Additionally, comparing floral temperatures between members of the same plant communities that do and do not display petal movement would help to elucidate the role of floral opening and closure in the modification of floral temperature.

## FLOWER PUBESCENCE

Pubescence is thought to be important for temperature modification of flowers in some species, but, as opposed to leaf pubescence, the ecophysiological functions of flower pubescence have been little studied. Thick leaf pubescence can heat leaves in cool, high-elevation environments by increasing the boundary layer of air adjacent to the leaf and reducing convective heat loss (Meinzer and Goldstein, 1985). Floral pubescence can provide a similar insulating effect.

The idea that pubescent flowers or inflorescences function as ‘hairy heat traps’ to warm floral structures was for example recognized in willows (Krog, 1955). In Alaska, woolly willow catkins can be up to 15–25 °C warmer with ambient air temperatures of 0 °C, and removal of pubescence reduces temperatures by about 60 % relative to unmanipulated catkins (Krog, 1955). In willows, sex-based differences in pubescence underlie disparities in inflorescence temperature with pistillate catkins having denser pubescence and, on average, being significantly warmer than staminate catkins (Kevan, 1989). Outside of alpine willows, the impact of inflorescence pubescence on floral temperature has been studied in a few low-latitude alpine species. In the Andes, species in the genus *Puya* (Bromeliaceae) from high elevations produce denser pubescence than those occurring at lower elevations (Miller, 1986). More glabrous low-elevation taxa tended to track ambient temperatures while high-elevation pubescent taxa tended to be warmer than ambient conditions. Control plants of *Puya hamate* maintained temperatures 2–3 °C higher than ambient night-time conditions while those denuded of pubescence did not. Finally, north-facing (warmer) inflorescences had elevated seed production compared to south-facing (cooler) inflorescences, providing a link between temperature and fecundity (Miller, 1986).

The thermal dynamics of inflorescences with respect to pubescence have been studied in two Himalayan taxa. Although the woolly inflorescence of *Saussurea medusa* (Asteraceae) is 5.9 °C warmer than ambient air temperatures, it is unlikely to be due to pubescence, but rather the compact architecture of the inflorescence itself (Yang *et al.*, 2008). Removal of pubescence *in situ* and in controlled conditions had negligible impact on heat retention, and thus Yang *et al.* (2008) posited that pubescence functions to repel water and/or to reflect UV light. In the Himalayan mint *Eriophyton wallichii* (Lamiaceae), flowers are covered by densely pubescent leaves. Pubescent control leaves absorb slightly more visible light than experimentally shaved leaves, and consequently are significantly warmer (Peng *et al.*, 2015). The authors evaluated pollen viability and seed production in control plants and those with experimentally lifted leaves, but from these treatments it is difficult to assess the direct effect of pubescence on fitness. Together these studies in Himalayan taxa suggest that pubescence may be less important than leaf architecture in mediating floral temperatures.

To date, only one study has evaluated the impact of pubescence on the thermal dynamics of reproductive structures and the downstream impacts on plant reproductive fitness (Miller, 1986). It is thus clear that additional studies to generalize the impact of pubescence are needed. Taken together, it appears that the ecological role of pubescence on the microclimate of the flower may be dynamic and habitat-specific because pubescence can impact temperature, water relationships and UV irradiance.

## ENDOGENOUS HEATING

Endogenous heating, or thermogenesis, is the process by which plants generate heat via metabolic processes. Endogenous heating of flowers occurs in at least 11 angiosperm families (reviewed by Thien *et al.*, 2000; Luo *et al.*, 2010), suggesting it has evolved several times independently. Some well-known genera that endogenously heat floral organs include *Arum*, *Nelumbo* (sacred lotus), *Philodendron* and *Victoria*. In addition to generating heat, some thermogenic flowers are remarkably constant in their temperature over several days despite changing ambient temperatures (Seymour and Schultze-Motel, 1996). Skunk cabbage (*Symplocarpus renifolius*), for example, maintains a constant 23 °C temperature, even at ambient temperatures of −10 °C (Seymour *et al.*, 2009). Experiments showed that pollen germination and pollen tube growth decline rapidly with changes from 23 °C (Seymour *et al.*, 2009), but whether thermogenesis evolved to match optimal pollen temperatures, vice versa, or both remains difficult to infer. Thermogenesis does not appear to have evolved as an adaptation for elevated pollen fertility in all species. In *Illicium* (Schisandraceae) flowers for example, endogenous heat production is highest well after sexual function. In this species heating benefits larval development of gall midges, which are the plant’s prime pollinator (Luo *et al.*, 2010).

Plant thermogenesis has been thoroughly investigated because of its specific physiological basis, such as its oxygen consumption (e.g. Nagy *et al.*, 1972; Seymour, 2010; Seymour *et al.*, 2015), but we know little about the floral traits that maintain endogenously produced heat. Thermogenic flowers are generally large, presumably because a low surface to volume ratio decreases heat loss and therefore thermogenesis is only effective from a certain minimal size (Seymour and Schultze-Motel, 1997). Given that many floral thermogenic species are pollinated by flies and beetles that copulate inside the flower, it has been hypothesized that thermogenesis is an adaptation to pollination by these groups (Seymour and Schultze-Motel, 1997; Thien *et al.*, 2000), although formal tests of this hypothesis are lacking.

Although the benefits of higher flower temperatures are often similar between thermogenesis and exogenous heat capture, thermogenesis provides a unique benefit. It allows flowers to heat up independently from ambient solar conditions, such as under dense vegetation or when the intensity of the sun is insufficient to elevate flower temperatures. It is currently difficult to make general conclusions as to the phylogenetic distribution and life history of flower thermogenesis because of its relative rareness. Few studies link the magnitude of endogenous heating with fitness parameters, which hinders drawing general conclusions and predicting why it is frequent in some groups. It would, for example, be interesting to know whether there is marked variation in endogenous heating among individuals or populations, and, if so, what (physiological) aspects cause this variation and how this variation ultimately translates to natural selection.

## GENERAL DISCUSSION

Plants occur in a vast range of climatic and ecological conditions, and given their sessile nature, it is unsurprising that plants have evolved floral traits that modify the temperature of their reproductive organs. Modification of floral temperature is likely to be crucial for plant reproduction in an ever-changing thermal environment under global change. We suggest the following steps be taken to advance our understanding of the thermal ecology of flowers. (1) Form–function studies must identify the floral traits that underlie variation in heat capture. (2) Investigations must determine how variation in floral temperature directly impacts reproductive performance via pollen and ovule performance, and through the impact of floral temperature on plant–animal interactions (e.g. pollination). (3) Comparative studies must evaluate how the functional traits that impact floral temperature co-vary with ecological niche and climatic region. In this review, we have discussed six floral traits that contribute to the floral heat budget and are strong candidates for future research on the thermal ecology of flowers.

Studies that investigate how floral heat capture influences plant fitness are scarce but are greatly needed. Adaptations to increase floral temperature are expected to be prominent in early blooming species and species that occur in extreme habitats, such as at high latitudes or altitudes. Indeed, heliotropism has been suggested to occur more when ambient temperatures are below optimal (e.g. Zhang *et al.*, 2010), but formally testing if adaptations to increase heat capture and/or retention occur more in colder climates requires broader species sampling. Translocation experiments may help to elucidate whether heat-capture mechanisms are local adaptations to low or highly variable thermal environments. Identification of systems with intraspecifc variation in floral temperature will be ideal for understanding natural selection. Additionally, large-scale comparative studies associating floral heat-capture mechanisms with habitat and abiotic parameters will be crucial for understanding agents of selection that shape diversity in floral thermal adaptations.

The perianth has largely been considered a communication signal to attract pollinators, but it also plays a major role in regulating the thermal environment of the flower. Petals can shield the reproductive organs from wind and rain, focus incident light on the reproductive organs, convert light to radiation and/or mediate heliotropism, but understanding the relative importance of these different functions is pivotal in understanding how natural selection shaped these features. Tethering, shading and removal of the perianth (e.g. Kevan, 1970; Kjellberg *et al.*, 1982) can help to elucidate how the perianth impacts floral temperature, but more detailed studies are required to infer their importance. By the same token, there may be trade-offs between different physiological and morphological aspects of flowers. As an example, in the case of colour polymorphic flowers where the reproductive organs are enclosed by the perianth (e.g. *Anthirrhinum*): are paler flowers warmer because they are more translucent and thus feature stronger microgreenhouse effects, or are darker flowers warmer owing to conversion of light to heat? Furthermore, how does this impact pollen viability, ovule viability and pollinator visitation?

Little is known about common physiological and optical properties of flowers with respect to their role in modification of floral temperature. As an example, the reflectivity and translucency of floral elements are crucial aspects of the flower’s thermal ecology. For example, the fraction of reflected light versus the fraction of transmitted light (van der Kooi *et al.*, 2016) is likely to shape intrafloral warming (Herrera, 1995; McKee and Richards, 1998). Although studies on some extreme cases involved more detailed physiology (see ‘Flower Morphology’), these cases are so exceptional and species-specific that their findings are difficult to extrapolate to other taxa. Similarly, retention of heat is probably higher for densely packed floral organs, such as inflorescences of composite flowers, compared to very loosely arranged organs.

Although some flower shapes, movements or anatomies may influence the thermal environment of the flower, one should bear in mind that this may not be the sole function of that property. For example, tubular flowers may exhibit microgreenhouse-like effects, but they may also function to provide a specific mechanical fit to pollinators and/or to exclude antagonists. Glossy flower surfaces may increase light reflection to the reproductive organs, but whether glossiness also plays a role in the visibility to pollinators remains unknown (reviewed by van der Kooi *et al.*, 2019). By the same token, flower orientation (e.g. heliotropism) may be important for temperature regulation, but it could also enhance the flower’s accessibility (Fenster *et al.*, 2009) and/or visibility to pollinators, such as when the sun-facing (adaxial) side features the strongest visual signal (Stavenga and van der Kooi, 2016). Indeed, in the Mexican cactus *Myrtillocactus geometrizans*, southward-facing flowers exhibit higher reproductive capacity than northward-facing flowers, but this is attributed to increased photosynthesis on the southward-facing parts of the plant (Aguilar-García *et al.*, 2018), and not to increase flower temperature.

Our review deals largely with the mechanisms of floral warming as opposed to floral cooling so tackles only one aspect of floral thermoregulation. Yet, floral cooling in particularly warm environments could enhance plant reproductive fitness, especially because pollen viability often declines at extremely high temperatures. The bias towards warming in this review reflects a geographical bias in the field of floral thermal ecology – the vast majority of work on floral thermal ecology has been conducted in alpine or arctic species. We encourage future studies on potential modes of floral cooling (e.g. evapotranspiration, convective heat loss), and the impact of floral temperatures that lower ambient conditions on pollinator visitation and reproductive success.

We conclude that the thermal ecology of flowers is an exciting topic that offers ample opportunity for future research. We welcome future multidisciplinary studies on the combined effects of different key factors such as floral shape, orientation and opening–closure (e.g. Yon *et al.*, 2017; Haverkamp *et al.*, 2019), on flower temperature, pollinator visitation and ultimately plant fitness.

## SUPPLEMENTARY DATA

Supplementary data are available online at https://academic.oup.com/aob and consist of the following. Table S1: Plant species for which floral heliotropism was described and, if studied, its functional significance.

mcz073_suppl_Supplementary_Material

## FUNDING

CJvdK was supported by a Veni grant (number 016.Veni.181.025) financed by the Netherlands Organisation for Scientific Research (NWO), MHK by the National Science Foundation (grant number DEB 1754590) and PGK by an NSERC Discovery Grant and a grant from the Canadian Ornamental Horticultural Association.
