# Supplementary material for: The thermal ecology of flowers
Source: Ann Bot. 2019 Jun 17;124(3):343–53. doi: 10.1093/aob/mcz073 (PMC6798827; doi:10.1093/aob/mcz073)
Supplement: mcz073_suppl_Supplementary_Material [file mcz073_suppl_supplementary_material.docx]

Table S1. Plant species for which floral heliotropism was described and, if studied, its functional significance.

| **Family** | **Name** | **Effect on flower biology** | **Study location** | **Reference** |
| --- | --- | --- | --- | --- |
| Asteraceae | *Oritropium limnophilum* | Heliotropism leads to warmer flowers and increases the amount of visits by insects. | Venezuelan mountains | (Smith 1975) |
| Asteraceae | *Callendula arvensis* | Heliotropism leads to warmer flowers and flower-basking insects. | Spain | (Orueta 2002) |
| Asteraceae | *Tragopogon dubius* | Flowers exhibit some heliotropism at least part of the day, but its functional significance is unknown. | US, Canada, Europe | (Kevan 2013) |
| Asteraceae | *Senecio congestus* | Flowers exhibit some heliotropism at least part of the day, but its functional significance is unknown. | Arctic | (Kevan 1972) |
| Asteraceae | *Leucanthemopsis alpina* | Flowers are warmer than ambient temperatures, but the effect of heliotropism is unclear. | Austrian Alps | (Luzar and Gottsberger 2001) |
| Asteraceae | *Matricaria ambigua* | Flowers exhibit some heliotropism throughout the day, but its functional significance unknown. | Arctic | (Kevan 1972) |
| Caryophyllaceae | *Cerastium alpinum* | Flowers are warmer than ambient temperatures, but the effect of heliotropism is unclear. | Arctic | (Hocking 1968) |
| Melanthiaceae | *Trillium nivale* | Flowers are warmer than ambient temperatures, but the effect of heliotropism is unclear. | Unknown | (McKee and Richards 1998) |
| Papaveraceae | *Papaver radicatum* | Heliotropism leads to warmer flowers and flower-basking insects. | Arctic | (Kevan 1972, 1975) |
| Ranunculaceae | *Adonis ramosa* | Heliotropism leads to warmer flowers and increases the amount of visits by insects. | Japan | (Kudo 1995) |
| Ranunculaceae | *Adonis nemorosa* | Flowers exhibit some heliotropism at least part of the day, but its functional significance is unknown. | Czechia | (Rejšková et al. 2010) |
| Ranunculaceae | *Anemone parviflorum* | Flowers exhibit some heliotropism at least part of the day, but its functional significance is unknown. | Arctic | (Kevan 2013) |
| Ranunculaceae | *Anemone rivularis* | Heliotropism leads to warmer flowers. | Chinese mountains | (Zhang et al. 2010) |
| Ranunculaceae | *Callianthemum coriandrifolium* | Flowers are warmer than ambient temperatures, but the effect of heliotropism is unclear. | Austrian Alps | (Luzar and Gottsberger 2001) |
| Ranunculaceae | *Hepatica* sp*.* | Flowers exhibit some heliotropism at least part of the day; functional significance unknown. | Unknown | (McKee and Richards 1998) |
| Ranunculaceae | *Pulsatilla alpina* | Flowers are warmer than ambient temperatures, but the effect of heliotropism is unclear. | Austrian Alps | (Luzar and Gottsberger 2001) |
| Ranunculaceae | *Ranunculus adoneus* | Heliotropism leads to warmer flowers and increases visits by insects, but comes with a water cost. | Colorado Rockies | (Stanton and Galen 1989; Galen 2006) |
| Ranunculaceae | *Ranunculus acris* | Heliotropic flowers are warmer, but heliotropism does not increase visitation by insects and has no effect on reproductive success | Southwest Norway | (Totland 1996) |
| Ranunculaceae | *Ranunculus alpestris* | Flowers are warmer than ambient temperatures, but the effect of heliotropism is unclear. | Austrian Alps | (Luzar and Gottsberger 2001) |
| Ranunculaceae | *Ranunculus montanus* | Heliotropism leads to warmer flowers and increases the amount of visits by insects. | Austrian Alps | (Luzar and Gottsberger 2001) |
| Rosaceae | *Dryas integrifolia* | Heliotropism leads to warmer flowers and flower-basking insects. | Arctic | (Hocking 1968; Kevan 1972, 1975) |
| Rosaceae | *Dryas octopetala* | Heliotropism leads to warmer flowers and flower-basking insects. | Arctic | (Kevan 1975; Kjellberg et al. 1982) |
| Violaceae | *Viola pedunculata* | Flowers exhibit some heliotropism at least part of the day; functional significance unknown. | Unknown | (Kutschera and Briggs 2015) |

**References**

Galen C (2006) Solar furnaces or swamp coolers: costs and benefits of water use by solar-tracking flowers of the alpine snow buttercup, Ranunculus adoneus. Oecologia 148:195–201. doi: 10.1007/s00442-006-0362-y

Hocking B (1968) Insect-flower associations in the high Arctic with special reference to nectar. Oikos 19:359–387

Kevan PG (2013) Mythical Clytie’s gyrations still spin at the top of the world. J. Pollinat. Ecol. (Magazine Sect. 1–4

Kevan PG (1972) Heliotropism in some Arctic flowers. Can Field-Naturalist 86:41–44

Kevan PG (1975) Sun-tracking solar furnaces in high arctic flowers: significance for pollination and insects. Science (80- ) 189:723–726

Kjellberg B, Karlsson S, Kerstensson I (1982) Effects of heliotropic movements of flowers of Dryas octopetala L. on gynoecium temperature and seed development. Oecologia 54:10–13

Kudo G (1995) Ecological significance of flower heliotropism in the spring ephemeral Adonis ramosa (Ranunculaceae). Oikos 72:14–20

Kutschera U, Briggs WR (2015) Phototropic solar tracking in sunflower plants: an integrative perspective. Ann Bot 117:1–8

Luzar N, Gottsberger G (2001) Flower heliotropism and floral heating of five alpine plant species and the effect on flower visiting in Ranunculus montanus in the Austrian Alps. Arctic, Antarct Alp Res 33:93–99

McKee J, Richards AJ (1998) Effect of flower structure and flower colour on intrafloral warming and pollen germination and pollen‐tube growth in winter flowering Crocus L. (Iridaceae). Bot J Linn Soc 128:369–384

Orueta D (2002) Thermal relationships between Calendula arvensis inflorescences and Usia aurata bombyliid flies. Ecology 83:3073–3085

Rejšková A, Brom J, Pokorný J, Korečko J (2010) Temperature distribution in light-coloured flowers and inflorescences of early spring temperate species measured by Infrared camera. Flora - Morphol Distrib Funct Ecol Plants 205:282–289. doi: 10.1016/J.FLORA.2009.05.001

Smith AP (1975) Insect pollination and helioptropism in Oritrophium limnophilum (Compositae) of the Andean Paramo. Biotropica 7:284–286

Stanton ML, Galen C (1989) Consequences of flower heliotropism for reproduction in an alpine buttercup (Ranunculus adoneus). Oecologia 78:477–485

Totland O (1996) Flower heliotropism in an alpine population of Ranunculus acris (Ranunculaceae): effects on flower temperature, insect visitation, and seed production. Am J Bot 83:452–458

Zhang S, Ai H-L, Yu W-B, et al (2010) Flower heliotropism of Anemone rivularis (Ranunculaceae) in the Himalayas: effects on floral temperature and reproductive fitness. Plant Ecol 209:301–312
